# Associations Between Adverse Childhood Experiences and Prenatal Depression Mediated Through Family Communication in Chinese Pregnant Women: Causal Mediation Analysis

**DOI:** 10.1155/da/3386991

**Published:** 2026-06-29

**Authors:** Yuehui Wen, Yihan Li, Yumeng Li, Mingyao Liu, Ningyuan Guo

**Affiliations:** ^1^ School of Nursing, Shanghai Jiao Tong University, Shanghai, China, sjtu.edu.cn; ^2^ College of Health Science and Technology, Shanghai Jiao Tong University School of Medicine, Shanghai, China, shsmu.edu.cn; ^3^ School of Public Health, Shanghai Jiao Tong University, Shanghai, China, sjtu.edu.cn; ^4^ Shanghai Jiao Tong University School of Medicine, Shanghai, China, shsmu.edu.cn

**Keywords:** adverse childhood experiences, causal mediation, family communication, pregnant women, prenatal depression

## Abstract

**Background:**

Adverse childhood experiences (ACEs) have shown impacts on prenatal depression. Most studies examined the cumulative risk of ACE scores, though impacts of different ACE types can vary. And less is known about the psychosocial mechanisms underlying the impacts. Supportive communication within a family formed through marriage may serve as a psychosocial resource for pregnant women.

**Objectives:**

To differentiate the associations of ACE scores and types with prenatal depression. The mediation effect of family communication was further investigated.

**Study Design:**

A cross‐sectional study in 873 Chinese pregnant women (mean age 30.89 [SD 3.93] years).

**Methods:**

ACEs, family communication, and prenatal depression were measured using the ACE International Questionnaire (ACE‐IQ), Family Communication Scale (FCS), and Edinburgh Postnatal Depression Scale (EPDS), respectively. Specifically, 13 types of ACEs included physical abuse, emotional abuse, sexual abuse, physical neglect, emotional neglect, domestic violence, parental death or separation, family substance use, family mental illness, family incarceration, bullying, collective violence, and community violence. Multivariable linear regression examined the associations of ACE scores and types with prenatal depression, adjusting for demographic, lifestyle, and pregnancy‐related covariates. Causal mediation analysis investigated the mediation effect of family communication.

**Results:**

Childhood abuse (physical, emotional, and sexual), neglect (physical and emotional), domestic violence, collective violence, and bullying showed associations with prenatal depression (adjusted *b* range 1.34–3.28, all *p*s <0.05). ACE scores were associated with prenatal depression (adjusted *b* = 0.55, 95% confidence interval [CI] 0.38, 0.72). Family communication mediated the associations of ACE scores (proportion of total effect [TE] mediated = 22%), physical (26%) and emotional abuse (15%), physical (57%) and emotional neglect (31%), and domestic violence (22%) with prenatal depression.

**Conclusions:**

Associations with prenatal depression were observed with ACE scores and were various across ACE types, which were medicated through family communication. Pregnant women with high ACE scores and specific ACE types can be vulnerable groups for prenatal depression, and interventions for promoting family communication are needed.

## 1. Introduction

Adverse childhood experiences (ACEs) that occurred before age 18 have been associated with poor health outcomes across individuals’ life course [1, 2], such as negative effects on neurodevelopment [3], chronic diseases [2], anxiety and depression [4], and increased healthcare utilization [5]. ACEs may be transmitted across generations, increasing the risk of exposure among the next generation [6]. The widely used ACE International Questionnaire (ACE‐IQ) categorized ACEs into 13 types: physical abuse, emotional abuse, sexual abuse, physical neglect, emotional neglect, domestic violence, parental death or separation, family substance use, family mental illness, family incarceration, bullying, collective violence, and community violence [7]. The prevailing method for studying the consequences of ACEs consists of summing individual events into cumulative scores [2]. However, using the cumulative ACE scores could mask the heterogeneity of diverse types of ACEs because this approach treats each ACE as equally contributing to study unfavorable outcomes [8] and limits understanding of the underlying mechanisms that link ACEs and mental health difficulties, thereby hindering the identification of specific targets for interventions [9].

Pregnancy is a critical period during which pregnant women experience substantial physiological, psychological, social, and familial changes, and these changes can contribute to emotional fluctuations and mental health problems [10, 11]. Prenatal depression has been one of the most serious problems of maternal mental health [12]. The prevalence of prenatal depression has reached 25.8% in China [13] and 20% worldwide [14]. Pregnant women have to face concerns about body image, fear of childbirth, and new parenting responsibilities, combined with hormonal fluctuations affecting the hypothalamic–pituitary–adrenal (HPA) axis and neurotransmitter systems, which may increase the vulnerability to depression [15]. A meta‐analysis showed that ACEs were positively associated with prenatal depression [4]. Compared to other mental consequences of ACEs, prenatal depression could impact both maternal and birth outcomes across generations, including complications of pregnancy and an increased risk of death in the first year of life [16–19].

Pregnant women exposed to ACEs can be at higher risk for prenatal depression [20]. The life course theory posited that early life experiences could set individuals on lasting health trajectories, being particularly sensitive to certain developmental periods, such as pregnancy [21]. It is a pivotal time in the lives of people with ACEs [22], which has increased medical and psychosocial risks as well as plasticity of both the maternal and fetal brains [23]. As women transition to parenthood and consider how they will care for their baby, the memories about ACEs may resurface, influencing their mental health and emotional well‐being [15]. Previous studies have illustrated that ACE scores had associations with prenatal depression [24]. The accumulation of multiple ACEs may increase the risk of future prenatal depression more than a single ACE did [25]. Therefore, assessing ACEs is crucial for pregnant women to identify vulnerable groups.

However, not all ACE types are equally impactful for prenatal mental health [26]. Few studies have disentangled the associations of different ACE types with prenatal depression [4]. A study in pregnant women found that higher levels of childhood abuse significantly predicted higher levels of depression symptoms during pregnancy [27]. Another study showed that only domestic violence remained significantly associated with prenatal high depressive symptoms, suggesting that not all ACE types contribute equally to prenatal depression [28]. Therefore, previous studies recommended taking an unpacking approach to ACE research, such as examining two types of ACEs (childhood abuse and household dysfunction) separately within the same statistical model rather than relying solely on cumulative scores [29].

Previous studies on mediating mechanisms generally concentrated on physiological and psychological factors at the individual level. For instance, systematic reviews of prenatal depression mechanisms have predominantly concentrated on biological pathways, such as cortisol dysregulation [30] and other psychological factors, including self‐esteem [31]. In fact, interpersonal factors, particularly family communication, can be a pivotal factor in mitigating adverse psychological outcomes for pregnant women [32]. ACE types, particularly those in relation to emotional abuse and emotional neglect, were suggested as strong predictors of social‐emotional outcomes in intimate partner relationships [33]. Conflict avoidance and emotional disengagement in the childhood can create enduring negative feedback loops for adult family communication, particularly when stressors like pregnancy trigger intergenerational cycles of maladaptive coping [34, 35]. Overall, ACEs could disrupt the acquisition of healthy communication skills, leading to persistent family communication deficits in adulthood that amplify psychological distress during pregnancy.

Family communication of a pregnant woman, which involved key household decision‐makers through marriage, such as husband/partner, parents, and in‐laws, was associated with better care practices during pregnancy [36]. Having more than usual arguments with the husband/partner was a risk factor for prenatal depression [37]. Supportive spousal communication during pregnancy may buffer the impact of childhood family dysfunction by providing new models of relational security [38]. Conversely, pregnant women living with both parents and parents‐in‐law, who had low‐quality communication and relationships within the extended family network, were associated with an increased risk of depressive symptoms [39]. However, less is known about the mediating role of family communication.

This study aimed to differentiate the associations of ACE scores and types with prenatal depression. Causal mediation analysis is based on the counterfactual framework of causal inference to explain a more complex causal mechanism compared to traditional mediation analysis [40]. Causal mediation analysis has been applied widely in psychology research to have a deeper understanding of how social factors affect the occurrence and development of depression [41, 42]. We thereby used causal mediation analysis to further investigate the potential mediation effects of family communication on the associations of ACE scores and types with prenatal depression.

## 2. Methods

### 2.1. Participants and Procedure

This cross‐sectional study was conducted in a tertiary maternity and gynecology specialty hospital in Shanghai, China, from March 2024 to November 2024. By using the convenience sampling method, participants were recruited with the following eligibility criteria: (1) aged ≥18 years; (2) no diagnosis of any psychiatric disorders; and (3) no severe physical conditions that impede interactions with nurses. Psychiatric disorders are established independent risk factors for prenatal depression that would confound the association between ACEs and the outcome under investigation [43]. Additionally, the presence of active psychiatric conditions may affect the reliability of self‐reported ACEs, as depression can negatively bias the recall and reappraisal of childhood experiences through transient cognitive distortions [44].

Trained nurses and students with medical and nursing backgrounds recruited pregnant women by distributing an invitation to the online survey in the hospital outpatient department. A short description of the study was presented on the first page of the survey. Implied consent to participate was indicated when participants responded to the survey items. The survey was anonymous and programmed to allow single completion per device to prevent duplicate submissions.

### 2.2. Measurements

#### 2.2.1. ACEs

ACE‐IQ was developed by the World Health Organization [7] and has been adapted into the simplified Chinese version [45]. The ACE‐IQ is a 35‐item measure that assesses exposure to 13 types of ACEs. Response options for each question were dichotomous (i.e., yes/no) based on a 5‐point Likert scale ranging from “Never” to “Always” or based on a 4‐point Likert scale ranging from “Never” to “Many times.” To calculate the ACE scores, we used the binary scoring method for ACEs whereby only “Never” was scored as “No,” and other affirmative responses were all scored as “Yes.” The total score ranges between 0 and 13, with a higher score indicating more ACEs [7]. Cronbach’s *α* of ACE‐IQ was 0.74 in the present study, with good internal consistency [46].

#### 2.2.2. Family Communication

The Family Communication Scale (FCS) is widely used to measure satisfaction with the aspects of positive communication among family members [47], which was adapted into the Chinese version [48]. FCS had 10 items on a 5‐point Likert scale ranging from 1 = “strongly disagree” to 5 = “strongly agree.” All items were summed to yield a total score of 10–50, with higher scores indicating better family communication. Cronbach’s *α* of FCS was 0.97 in the present study, with good internal consistency [46].

#### 2.2.3. Prenatal Depression

The Edinburgh Postnatal Depression Scale (EPDS) was used to screen for depression within the last 7 days [49]. EPDS had 10 items on a 4‐point Likert scale ranging from 0 to 3. The total score ranged between 0 and 30, with higher scores indicating higher levels of prenatal depression. Cronbach’s *α* of EPDS was 0.91 in the present study, with good internal consistency [46].

#### 2.2.4. Demographic, Lifestyle, and Pregnancy‐Related Characteristics

An investigator‐developed questionnaire was used to collect demographic, lifestyle, and pregnancy‐related characteristics. Demographic and lifestyle characteristics including age, BMI, educational attainment, marital status, monthly household income, smoking, secondhand smoke exposure at home, and drinking, were assessed. Gestational weeks, abnormal pregnancy‐labor history, methods of conception, pregnancy planning, prenatal checkup results, chronic diseases, and pregnancy complications were collected as pregnancy‐related characteristics.

### 2.3. Statistical Analysis

All variables were checked independently, with a skewness value of |2.0| and a kurtosis value of |7.0| indicating normality [50]. EPDS scores were compared among different demographic, lifestyle, and pregnancy‐related characteristics. We examined the assumption of equal variance between groups and chose methods to test for equal variances or unequal variances. Characteristics were selected as pretreatment covariates if showing significant differences in equal variances in two‐sample *t* tests, one‐way analysis of variance (ANOVA), or Kruskal–Wallis test. Pearson correlation analysis was used to test the correlation among age, BMI, and prenatal depression. Associations of ACE scores and types with prenatal depression were examined using multivariable linear regression analyses, adjusting for demographic, lifestyle, and pregnancy‐related covariates. Unstandardized (*b*s) and standardized (*β*s) linear regression coefficients were reported, with *β* <0.2, 0.2–0.5, ≥0.5 indicating small, moderate, and large effect sizes, respectively [51].

We followed standard causal mediation analysis procedures [52]: (1) fitted the mediator and outcome models, (2) used the R package “mediate” to estimate the average causal mediation effect (ACME), and (3) applied the R package “medsens” to conduct sensitivity analysis for unobserved pretreatment confounders affecting both the mediator and outcome.

ACME, average direct effect (ADE), total effect (TE), and proportion of TE mediated (Prop.Mediated) can be estimated based on the two fitted models using nonparametric bootstrap. A significant mediation effect was inferred when the 95% confidence interval (CI) of the effect excluded zero. Sensitivity analysis was conducted to assess the robustness of the ACME to unobserved pretreatment confounders. The parameter “ρ” represented the correlation between the two error terms of the linear models for the mediator and outcome variables, which identified the degree of confounding at which the 95% CI of the ACME would include zero [53]. By calculating the ACME (point estimates and 95% CI) across a range of *ρ*‐values (from −1 to +1), we can evaluate how strong the unmeasured confounding would need to be to nullify the observed mediation effect. The higher the *ρ*‐value required to reduce the ACME to zero, the more robust the mediation effect is to unmeasured confounding [54]. A critical |*ρ*|≥0.20 was considered the minimum threshold for meaningful robustness [55]. $R_{M}^{*2}R_{Y}^{*2}$ is the product of coefficients of determination which represent the proportion of the previously unexplained variance in the mediator and outcome variables that is explained by an unobservable pretreatment unconfounder. And ${\tilde{R}}_{M}^{2}{\tilde{R}}_{Y}^{2}$ is in terms of the proportion of the original variance explained by an unobserved confounder. These analyses were conducted with the use of R software 4.3.2 [52]. Complete case analysis was used to handle missing data by excluding cases with missing values on any variables of interest. All measures in the present study were mandatory in the survey, and seven participants were excluded due to incomplete survey responses.

## 3. Results

Table 1 shows that of 873 participants, the mean age (SD) was 30.89 (3.93) years. 57.85% had completed university education, 79.96% had full‐time work, and 35.97% reported a monthly household income of 10,000–20,000 CNY (equivalent to 1379 −2759 USD). 98.51% of participants never smoked and 76.17% never drank, while 23.60% were exposed to secondhand smoke at home. The majority of participants had no pregnancy complications (74.46%) and no chronic diseases (97.37%). Significant differences in prenatal depression were observed for educational attainment, employment status, marital status, monthly household income, smoking, secondhand smoke exposure at home, drinking, gestational week, and pregnancy complications, which were selected as covariates.

**Table 1:** Demographic, lifestyle, and pregnancy‐related characteristics with Edinburgh Postnatal Depression Scale score (*N* = 873).

Characteristic	*n* (%)/mean ± SD	EPDS score (mean ± SD)^a^	*p* ^b^
Age (years)	30.89 ± 3.93	—	<0.001^c^
BMI	24.56 ± 4.60	—	0.24
Educational attainment			0.02
High school or below	57 (6.53)	7.86 ± 5.15	—
Junior college	161 (18.44)	7.94 ± 6.42	—
University	505 (57.85)	6.35 ± 6.04	—
Postgraduate or above	150 (17.18)	6.08 ± 5.44	—
Employment status			<0.001
Full time	698 (79.96)	6.01 ± 5.47	—
Part time	36 (4.12)	11.25 ± 6.53	—
Self‐employed	49 (5.61)	7.37 ± 6.69	—
Unemployment/full‐time student/volunteer/housekeeper	90 (10.31)	10.03 ± 6.60	—
Marital status			0.03
Married	853 (97.71)	6.67 ± 5.92	—
Unmarried	20 (2.29)	9.70 ± 7.64	—
Monthly household income (RMB, USD 1 = RMB 7.26)			<0.001
<10,000	125 (14.32)	8.29 ± 6.43	—
10,000–20,000	314 (35.97)	7.86 ± 6.12	—
20,000–30,000	245 (28.06)	6.19 ± 5.87	—
≥30,000	189 (21.65)	4.76 ± 4.75	—
Smoking			<0.001
Never	860 (98.51)	6.64 ± 5.88	—
Ever	13 (1.49)	13.00 ± 7.82	—
Secondhand smoke exposure at home			<0.001
Yes	206 (23.60)	6.05 ± 5.72	—
No	667 (76.40)	8.07 ± 5.72	—
Drinking			<0.001
Never	665 (76.17)	5.97 ± 5.62	—
Ever	191 (21.88)	8.63 ± 6.14	—
Currently	17 (1.95)	9.53 ± 6.56	—
Gestational week	26.16 ± 11.38	—	<0.001
<14	287 (32.87)	8.41 ± 6.54	—
14–28	79 (9.05)	8.48 ± 6.63	—
≥29	507(58.08)	5.51 ± 5.10	—
Abnormal pregnancy‐labor history			0.94
Yes	122 (13.97)	7.03 ± 6.24	—
No	751 (86.03)	6.75 ± 5.66	—
Methods of conception			0.49
Natural conception	827 (94.73)	6.95 ± 6.12	—
Assisted reproduction	46 (5.27)	6.20 ± 5.53	—
Pregnancy planning			0.42
Planned pregnancy	462 (52.92)	6.77 ± 6.12	—
Unplanned pregnancy	114 (13.06)	7.56 ± 6.36	—
Trying to conceive naturally	297 (34.02)	6.54 ± 5.54	—
Prenatal checkup results			0.09
Normal	773 (88.54)	7.06 ± 6.13	—
Abnormal	100 (11.46)	5.83 ± 5.80	—
Chronic diseases			0.72
Yes	23 (2.63)	6.35 ± 4.42	—
No	850 (97.37)	6.79 ± 6.00	—
Pregnancy complications			0.005
Yes	223 (25.54)	5.81 ± 5.25	—
No	650 (74.46)	7.25 ± 6.28	—

*Note*: Edinburgh Postnatal Depression Scale, range 0–30.

Abbreviations: M, mean; SD, standard deviation.

^a^Mean EPDS score (SD) = 6.78 (5.97).

^b^Age and BMI were used Pearson correlation analysis. Marital status, smoking, secondhand smoke exposure at home, abnormal pregnancy‐labor history, methods of conception, prenatal checkup results, chronic diseases, and pregnancy complications were used independent *t*‐test. With equal‐variances, educational attainment, drinking, and pregnancy planning were used one‐way analysis of variance (ANOVA). With unequal‐variances, employment status, monthly household income, and gestational weeks were used Kruskal–Wallis test.

^c^Age had moderate and negative correlation with prenatal depression (*r* = −0.21).

**Figure 1:**
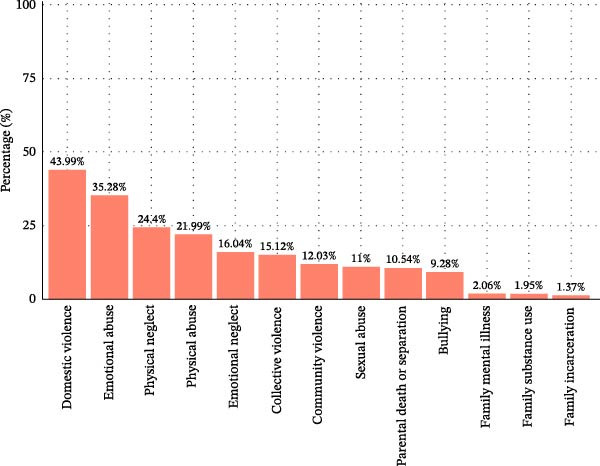
Bar chart depicting the prevalence of different types of ACEs among participants.

Figure 1 presents the prevalence of 13 ACE types among participants. Five ACE types exceeded 15% prevalence: domestic violence (43.99%), emotional abuse (35.28%), physical neglect (24.40%), emotional neglect (16.04%), and collective violence (15.12%). Moderate prevalence (10%–15%) was observed for community violence (12.03%), sexual abuse (11.00%), and parental death or separation (10.54%). The remaining five ACE types showed low prevalence (<10%): bullying (9.28%), family mental illness (2.06%), family substance use (1.95%), and family incarceration (1.37%).

Table 2 shows that ACE scores demonstrated an association with prenatal depression after adjusting for covariates (adjusted *b* = 0.55, 95% CI 0.38, 0.72, *β* = 0.20). Among specific ACE types, physical abuse (adjusted *b* = 2.10, 95% CI 1.18, 3.01, *β* = 0.14), emotional abuse (adjusted *b* = 2.40, 95% CI 1.63, 3.16, *β* = 0.19), sexual abuse (adjusted *b* = 1.38, 95% CI 0.17, 2.58, *β* = 0.07), physical neglect (adjusted *b* = 1.34, 95% CI 0.46, 2.24, *β* = 0.10), emotional neglect (adjusted *b* = 2.38, 95% CI 1.35, 3.80, *β* = 0.15), domestic violence (adjusted *b* = 1.57, 95% CI 0.82, 2.32, *β* = 0.13), bullying (adjusted *b* = 3.28, 95% CI 1.98, 4.57, *β* = 0.16), collective violence (adjusted *b* = 1.69, 95% CI 0.65, 2.73, *β* = 0.10) had significant associations with prenatal depression after adjusting for covariates (all *ps* < 0.05). Family substance use, parental death or separation, family mental illness, family incarceration, and community violence didn’t show significant associations after covariate adjustment.

**Table 2:** Associations of ACE scores and types with prenatal depression.

ACEs	Unadjusted association		Adjusted association^a^	
	*b* (95% CI)	*β*	*b* (95% CI)	*β*
ACE scores	0.64 (0.46, 0.81) ^∗∗∗^	0.23	0.55 (0.38, 0.72) ^∗∗∗^	0.20
ACE types				
Physical abuse	1.44 (0.49, 2.39) ^∗∗^	0.10	2.10 (1.18, 3.01) ^∗∗∗^	0.14
Emotional abuse	2.65 (1.84, 3.46) ^∗∗∗^	0.41	2.40 (1.63, 3.16) ^∗∗∗^	0.19
Sexual abuse	2.41 (1.15, 3.67) ^∗∗∗^	0.64	1.38 (0.17, 2.58) ^∗^	0.07
Physical neglect	2.37 (1.47, 3.28) ^∗∗∗^	0.46	1.34 (0.46, 2.24) ^∗∗^	0.10
Emotional neglect	2.27 (1.20, 3.34) ^∗∗∗^	0.55	2.38 (1.35, 3.40) ^∗∗∗^	0.15
Domestic violence	1.23 (0.44, 2.03) ^∗∗^	0.10	1.57 (0.82, 2.32) ^∗∗∗^	0.13
Family substance use	0.34 (−2.53, 3.21)	0.01	−2.72 (−5.58, 0.12)	−0.06
Parental death or separation	0.26 (−1.04, 1.55)	0.01	0.12 (−1.12, 1.35)	0.01
Family mental illness	1.30 (−1.50, 4.09)	0.03	−0.79 (0.58, 1.97)	−0.19
Family incarceration	2.76 (−0.65, 6.16)	0.05	−0.83 (−4.30, 2.63)	−0.16
Bullying	4.21 (2.88, 5.55) ^∗∗∗^	0.21	3.28 (1.98, 4.57) ^∗∗∗^	0.16
Collective violence	2.43 (1.33, 3.52) ^∗∗∗^	0.15	1.69 (0.65, 2.73) ^∗∗∗^	0.10
Community violence	1.42(0.20, 2.63) ^∗^	0.08	1.06 (−0.08, 2.20)	0.06

^a^Adjusted for age, education attainment, employment status, marital status, monthly household income, smoking, drinking, secondhand smoke exposure at home, gestational weeks, and pregnancy complications.

^∗^ *p*  < 0.05.

^∗∗^ *p*  < 0.01.

^∗∗∗^ *p*  < 0.001.

**Figure 2:**
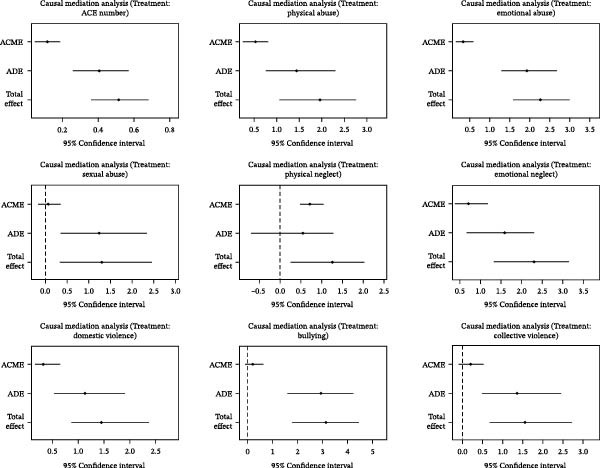
Graphical display of results from the mediate function.

Table 3 and Figure 2 show that ACE scores, physical abuse, emotional abuse, emotional neglect, and domestic violence had significant partial mediation effects on prenatal depression mediated through family communication (ACME range 0.11, 0.71; all *p*s <0.05). Emotional neglect had the largest partial mediation effect (ACME estimate= 0.71, *p* < 0.001) with 31% of mediated association (*p* < 0.001). Physical neglect had significant full mediation effects (ACME estimate = 0.71, *p* < 0.001; ADE estimate = 1.59, *p* > 0.05). Sexual abuse, bullying, and collective violence did not show significant mediation effects.

**Table 3:** Results of causal mediation analysis^a^.

Treatment	ACME		ADE		TE		Prop.Mediated	
	Estimate (95% CI)	*p*	Estimate (95% CI)	*p*	Estimate (95% CI)	*p*	Estimate (95% CI)	*p*
ACE scores	0.11 (0.06, 0.18)	<0.001	0.41 (0.26, 0.61)	<0.001	0.52 (0.36, 0.72)	<0.001	0.22 (0.12, 0.36)	<0.001
ACE type								
Physical abuse	0.52 (0.28, 0.83)	<0.001	1.44 (0.63, 2.33)	<0.001	1.96 (1.00, 2.79)	<0.001	0.26 (0.14, 0.46)	<0.001
Emotional abuse	0.33 (0.14, 0.61)	<0.001	1.93 (1.17, 2.65)	<0.001	2.27 (1.44, 3.01)	<0.001	0.15 (0.06, 0.29)	<0.001
Sexual abuse	0.06 (−0.24, 0.37)	0.84	1.24 (0.23, 2.31)	0.02	1.30 (0.10, 2.44)	0.02	0.04 (−1.08, 0.34)	0.82
Physical neglect	0.71 (0.44, 1.03)	<0.001	0.54 (−0.31, 1.67)	0.28	1.25 (0.34, 2.27)	<0.001	0.57 (0.25, 1.86)	<0.001
Emotional neglect	0.71 (0.41, 1.07)	<0.001	1.59 (0.14, 2.62)	0.02	2.30 (0.87, 3.49)	<0.001	0.31 (0.18, 0.85)	<0.001
Domestic violence	0.32 (0.13, 0.61)	<0.001	1.13 (0.37, 1.91)	<0.001	1.45 (0.67, 2.16)	<0.001	0.22 (0.08, 0.51)	<0.001
Bullying	0.21 (−0.08, 0.57)	0.18	2.93 (1.76, 4.33)	<0.001	3.14 (1.85, 4.59)	<0.001	0.07 (−0.04, 0.20)	0.18
Collective violence	0.19 (−0.12, 0.45)	0.28	1.35 (0.49, 2.36)	<0.001	1.55 (0.56, 2.67)	<0.001	0.13 (−0.14, 0.33)	0.28

*Note*: ACME, the average causal mediation effect; Prop.Mediated, proportion of total effect mediated.

Abbreviations: ADE, average direct effect; CI, confidence interval; TE, total effect.

^a^Including the observed pretreatment covariates: age, education attainment, employment status, marital status, monthly household income, smoking, drinking, secondhand smoke exposure at home, gestational weeks, and pregnancy complications.

Table S1 reports mean scores (SD) of ACEs‐IQ, EPDS, and FCS of 2.04 (0.07), 6.80 (0.20), and 21.90 (0.30), respectively. Table S2 shows that in sensitive analysis, ACEs scores, physical abuse, emotional abuse, emotional neglect, and domestic violence were all robust to potential unmeasured mediator‐outcome confounding, where a moderate confounder (*ρ* = 0.25) could turn ACMEs of these treatments to zero (i.e., all 95% CIs of ACME contain zero).

## 4. Discussion

Our study showed that ACE scores had an association with prenatal depression. Associations varied among different ACE types. Causal mediation analysis showed that ACE scores (proportion of TE mediated = 22%), physical abuse (26%), emotional abuse (15%), emotional neglect (31%), and domestic violence (22%) had significant partial mediation effects on prenatal depression mediated through family communication. Physical neglect had significant full mediation effects (57%).

ACE scores and certain ACE types were associated with prenatal depression. Previous studies have underscored the importance of considering the emotional domain of childhood experiences in the context of maternal mental health [56]. In this study, ACE scores were associated with prenatal depression, which aligned with the cumulative adversity hypothesis, which posited that the impact of ACEs on mental health outcomes is incremental [57]. Besides, among ACE types, childhood abuse (physical, emotional, and sexual), neglect (physical and emotional), domestic violence, bullying, and collective violence had stronger associations with prenatal depression. As previous studies showed, the long‐term effects of childhood trauma, including emotional abuse, neglect, and bullying, created a heightened sensitivity to stress during pregnancy, making these experiences significant risk factors for prenatal depression [58]. Emotional abuse demonstrated the strongest association with prenatal depression because of disruption in emotional regulation, self‐esteem, and coping mechanisms, predisposing individuals more vulnerable to mental health issues later in life [59]. Bullying demonstrated the second strongest association with prenatal depression. Child bullying can cause lasting damage to one’s self‐worth and social relationships [60], leaving individuals more susceptible to feelings of isolation and emotional distress during pregnancy.

We have noted that domestic violence has the association with prenatal depression. Domestic violence was defined as frequent exposure before age 18 to witness or experience physical violence or threats of violence between parents or adult household members, including acts such as pushing, grabbing, slapping, throwing objects, or hitting that caused marks or injury [7]. A previous study showed that parental divorce in childhood increased the risk for prenatal depression [61]. Witnessing domestic violence in childhood constitutes a direct interpersonal trauma that can induce lasting dysregulation of the HPA axis to develop the vulnerability to prenatal depression [62]. A recent study of 3126 Chinese female college students reported a 42.09% prevalence of childhood witnessing domestic violence [63]. Another study of 600 Chinese women reported that 31.67% were exposed to physical violence between their parents [64]. The prevalence of domestic violence exposure among Chinese women possibly varied across studies, which is needed to be more precisely evaluated in the future. Interestingly, other ACE types relevant to family members (i.e., family substance use, parental death or separation, family mental illness, and family incarceration) did not show significant associations with prenatal depression. This was consistent with previous results that among all family‐related ACEs, only domestic violence remained significantly associated with prenatal depression after adjustment [28]. Future research is warranted to further clarify the impacts and mechanisms of family‐related ACEs on prenatal mental health.

Our study also showed that family communication had a mediating effect on ACEs and prenatal depression. Poor family communication amplifies this negative impact. A study of Bangladeshi adolescents found that family support fully mediated the associations between ACEs and depression, while friend support showed smaller effects, highlighting the primacy of family‐level relational mechanisms [65]. A longitudinal study found that distinct ACE types differed in their associations with psychopathology and proposed mediators, with family‐level factors showing stronger links to social information processing deficits [66].

Notably, we observed variations in how different ACE types influence prenatal depression through family communication. The present study showed that emotional abuse and emotional neglect had particularly significant mediation effects on prenatal depression, which is consistent with the study of the long‐term impact of childhood emotional abuse on adult mental health [67] and with the persistent impact of childhood abuse on the long‐term effects of childhood adversity on adult mental health [68]. Although sexual abuse, bullying, and collective violence were associated with prenatal depression, they were not mediated by family communication. A previous study suggested sexual abuse could disrupt social functioning through distinct pathways, for example, of internalized shame and trust deficits, rather than specifically through family communication [66]. Future studies could further explore more effective mediating factors for sexual abuse and prenatal depression. Additionally, bullying and collective violence may operate primarily through peer‐ or community‐level relational pathways, such as peer rejection and school disaffection, beyond the scope of family communication systems [69].

## 5. Limitations

This study had several limitations. First, participants were recruited in a tertiary maternity and gynecology specialty hospital in Shanghai, restricting sample representativeness. Future research may include multiple hospitals, particularly across different rural or less affluent communities, to increase the external validity of our findings. Second, prenatal depression may vary across the pregnancy period. Longitudinal data are warranted to capture the temporal dynamics of variables. Third, ACEs were self‐reported retrospectively, which were subject to recall and social desirability biases. Future research can collect multisource data (e.g., corroborative data from childhood records or third‐party informants) to enhance the accuracy of the ACE measurement. Fourth, although our model included a range of covariates, it may have failed to account for important involved confounders, such as friendships, community resources, and quality of current partner relationships. Comprehensive assessments of social and physiological factors are called for in future research. Fifth, as a cross‐sectional study, the mediation effects identified in this study should be interpreted as significant mediation associations rather than a causal mediation effect. Future longitudinal research is warranted to further clarify and validate the causality of these relationships. Sixth, the measurement of ACEs assessed the occurrence of different ACE types rather than the frequency or duration of each experience. Future research could incorporate more nuanced assessments of ACEs, such as repeated exposure, to better characterize the cumulative risk of adversity.

## 6. Conclusions

Our study showed associations of ACE scores and types with prenatal depression in Chinese pregnant women. Child abuse (physical, emotional, and sexual), neglect (physical and emotional), domestic violence, collective violence, and bullying showed associations with prenatal depression. ACE scores had an association with prenatal depression. And associations of ACE scores, physical abuse, emotional abuse, physical neglect, emotional neglect, and domestic violence with prenatal depression were significantly mediated through family communication. Interventions for promoting family communication are needed for pregnant women with high ACE scores and certain ACE types.

## Author Contributions

**Yuehui Wen**: methodology, formal analysis, software, visualization, writing – original draft, writing – review and editing. **Yihan Li, Yumeng Li, and Mingyao Liu**: writing – original draft. **Ningyuan Guo**: conceptualization, project administration, supervision, data curation, validation, funding acquisition, writing – review and editing.

## Funding

This work was supported by the National Natural Science Foundation of China (Grant 82304261) and the Shanghai Science and Technology Development Foundation of “Rising Star” Sailing Program (Grant 23YF1421100).

## Ethics Statement

The studies involving human participants were reviewed and approved by Ethics committee of the International Peace Maternity and Child Health Hospital of China Welfare Institute affiliated with Shanghai Jiao Tong University School of Medicine (IRB Approval Number GKLW‐A‐2024‐086‐01). The patients/participants provided their written informed consent to participate in this study.

## Conflicts of Interest

The authors declare no conflicts of interest.

## Supporting Information

Additional supporting information can be found online in the Supporting Information section.

## Supporting information


**Supporting Information** Table S1. Mean scores of ACEs‐IQ, EPDS, and FCS (*N* = 873). Table S2. Results of sensitive analysis.

## Data Availability

The data sets generated for this study are available upon reasonable requests from the corresponding author.
