# Supplementary material for: Associations Between Adverse Childhood Experiences and Prenatal Depression Mediated Through Family Communication in Chinese Pregnant Women: Causal Mediation Analysis
Source: Depress Anxiety. 2026 Jun 29;2026:3386991. doi: 10.1155/da/3386991 (PMC13315836; doi:10.1155/da/3386991)
Supplement: Supplementary file 1 — Supporting Information Table S1. Mean scores of ACEs‐IQ, EPDS, and FCS (N = 873). Table S2. Results of sensitive analysis. [file DA-2026-3386991-s001.docx]

Table S1. Mean scores of ACEs-IQ, EPDS, and FCS (N=873).

|  | Range | Mean±SD |
| --- | --- | --- |
| ACE scores^a^ | 0-13 | 2.04±0.07 |
| Prenatal depression^b^ | 0-30 | 6.80±0.20 |
| Family communication^c^ | 10-50 | 21.90±0.30 |

Note. SD: standard deviation.

^a^ ACEs was measured using the ACE International Questionnaire (ACE-IQ), with higher scores indicating higher levels.

^b^ Prenatal depression was measured using the Edinburgh Postnatal Depression Scale (EPDS), with higher scores indicating higher levels.

^c^ Family communication was measured using the Family Communication Scale (FCS), with higher scores indicating higher levels.

Table S2. Results of sensitive analysis.

| Treatment | ρ | 95% CI of ACME | | $R_{M}^{*2}R_{Y}^{*2}$ | $\tilde{R}_{M}^{2}\tilde{R}_{Y}^{2}$ |
| --- | --- | --- | --- | --- | --- |
|  |  | Lower | Upper |  |  |
| ACE scores | 0.25 | -0.002 | 0.057 | 0.040 | 0.029 |
| ACE type |  |  |  |  |  |
| Physical abuse | 0.25 | -0.083 | 0.163 | 0.063 | 0.046 |
| Emotional abuse | 0.25 | -0.054 | 0.107 | 0.063 | 0.046 |
| Physical neglect | 0.25 | -0.106 | 0.230 | 0.063 | 0.046 |
| Emotional neglect | 0.25 | -0.129 | 0.215 | 0.063 | 0.046 |
| Domestic violence | 0.25 | -0.046 | 0.106 | 0.063 | 0.068 |

Note. ACEs scores had the least effect on the mediators and outcomes (${\tilde{\text{R}}}_{\text{M}}^{\text{2}}{\tilde{\text{R}}}_{\text{Y}}^{\text{2}}$ = 2.9%) caused from unobserved pretreatment confounders. In a word, the findings implied the significant mediation effects almost didn’t be affected by unobserved pretreatment confounders.


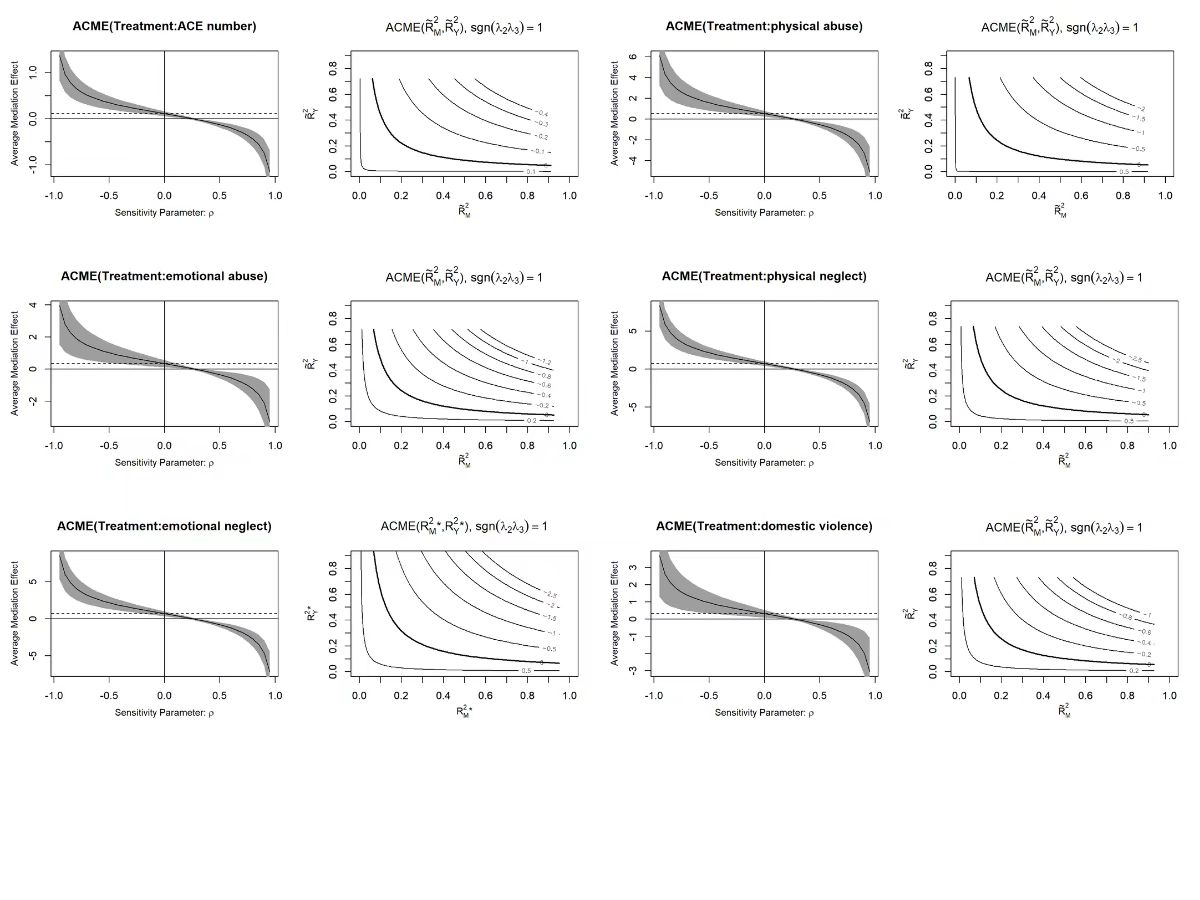


Figure S1. Sensitivity analysis regarding indirect effect with unmeasured confounders under different correlation (ρ) average causal mediation effect.

Note. The resulting “ρ” figures plot the estimated true values of ACME (or ADE, Prop.Mediated) against ρ, along with the 95% CI. When ρ is zero, sequential ignorability holds, so the estimated value at that point will be equal to the estimate returned by the mediate. The confidence level is determined by the “CI” value of the original mediate object. In the left-side of plots, the dashed horizontal line represents the estimated mediation effect under the sequential ignorability assumption, and the solid line represents the mediation effect under various values of ρ. The gray region represents the 95% confidence bands. In the right-hand side of plots, each contour line represents the mediation effect for the corresponding values of $\tilde{R}_{M}^{2}$ and $\tilde{R}_{Y}^{2}$ .
